# Inflammatory response of gut, spleen, and liver in mice induced by orally administered *Porphyromonas gingivalis*

**DOI:** 10.1080/20002297.2022.2088936

**Published:** 2022-06-16

**Authors:** Yingman Liu, Wenkai Huang, Ke Dai, Ni Liu, Jiaqi Wang, Xiaoying Lu, Jiaojiao Ma, Manman Zhang, Mengqi Xu, Xu Long, Jie Liu, Yurong Kou

**Affiliations:** a Department of Periodontics, School and Hospital of Stomatology, China Medical University, Liaoning Provincial Key Laboratory of Oral Diseases, Shenyang, Liaoning, China; b Department of Orthodontics, School of Stomatology, China Medical University, Shenyang, Liaoning, China; c Department of Stomatology, Lishui University School of Medicine, Lishui, Zhejing, China; d Department of Oral Biology, School and Hospital of Stomatology, China Medical University, Liaoning Provincial Key Laboratory of Oral Diseases, Shenyang, Liaoning, China; e Department of Stomatology, Science Experiment Center, China Medical University, Shenyang, Liaoning, China

**Keywords:** *Porphyromonas gingivalis*, periodontal disease, intestinal bacteria, inflammation, immune response

## Abstract

**Background:**

Periodontitis is a chronic multifactorial inflammatory disease. *Porphyromonas gingivalis* is a primary periopathogen in the initiation and development of periodontal disease. Evidence has shown that *P. gingivalis* is associated with systemic diseases, including IBD and fatty liver disease. Inflammatory response is a key feature of diseases related to this species.

**Methods:**

C57BL/6 mice were administered either PBS, or *P. gingivalis*. After 9 weeks, the inflammatory response in gut, spleen, and liver was analyzed.

**Results:**

The findings revealed significant disturbance of the intestinal microbiota and increased inflammatory factors in the gut of *P. gingivalis*-administered mice. Administrated *P. gingivalis* remarkably promoted the secretion of IRF-1 and activated the inflammatory pathway IFN-γ/STAT1 in the spleen. Histologically, mice treated with *P. gingivalis* exhibited hepatocyte damage and lipid deposition. The inflammatory factors IL-17a, IL-6, and ROR-γt were also upregulated in the liver of mice fed with *P. gingivalis*. Lee’s index, spleen index, and liver index were also increased.

**Conclusion:**

These results suggest that administrated *P. gingivalis* evokes inflammation in gut, spleen, and liver, which might promote the progression of various systemic diseases.

## Introduction

Periodontitis, an infectious disease that is prevalent worldwide, induces damage to the soft and hard tissues in the periodontium, which can eventually lead to tooth loss [1,2]. Bacterial infection and the subsequent immune response are the main causes of tissue destruction and bone loss in periodontitis. *Porphyromonas gingivalis*, a Gram-negative anaerobic bacterium, possessing a complex of virulence factors including gingipains, lipopolysaccharide (LPS), and capsular polysaccharides, is a primary periopathogen in the initiation and development of periodontitis. *P. gingivalis* is also deemed to be a risk factor for various diseases and conditions, including atherosclerosis [3], rheumatoid arthritis [4], non-alcoholic steatohepatitis [5], and Alzheimer’s disease [6]. *P. gingivalis* can also diffuse to distal sites or the circulation from local sites of infection [7]. Gastrointestinal and hematogenous routes are two principle paths through which oral bacteria can translocate. The gastrointestinal route is also recognized as a means by which oral bacterial can diffuse to the gut. Schmidt et al. found that a majority of oral bacteria can be transferred to the colorectum in healthy individuals and that higher levels of transmission occur in colorectal cancer (CRC) patients [8]. Anatomically, the mouth is the beginning of the gastrointestinal tract. Oral bacteria can thus transmigrate to the intestinal tract together with food, water, and saliva [9]. In line with this, periodontitis has been shown to be associated with gastrointestinal cancer and inflammatory bowel disease (IBD) [10,11]. Studies have also shown that the total bacterial load and microbial richness in the saliva of periodontitis patients are far higher than those of healthy individuals [12]. Additionally, according to quantitative analyses, patients with severe periodontitis swallow approximately 10^12^–10^13^ *P. gingivalis* bacteria per day [13–15]. *P. gingivalis* also can travel to the gut where it disrupts the intestinal environment [16,17]; the resulting imbalance of intestinal homeostasis can in turn lead to a series of systemic diseases. However, the effects that *P. gingivalis* exerts on the gastrointestinal tract have not been elucidated.

Strong anatomical and functional interactions are known to occur among the gut, spleen, and liver. The spleen, as the largest secondary lymphoid organ, possesses a wide array of immunological functions. Unlike other peripheral immune organs, the spleen lacks afferent lymphatics and hence all antigens and cells reach the spleen via the blood [18]. The surface of the human gastrointestinal tract is the primary site at which foreign antigens are sensed, leading to promotion of the local gut immune response. In the intestinal tract, antigens express their intestinal immunomodulatory effects first through interaction between antigens and immunocompetent cells in the intestinal mucosal immune system [19,20]. These gut-derived antigens possibly gain access to the systemic circulation, where they are endocytosed, processed, and presented by splenic dendritic cells to naïve T cells, promoting their transformation to colitogenic effector cells within the spleen [21]. Some studies have also shown that the spleen participates in the mucosal immune response in the gut [22,23]. Because intestinal and splenic blood flow forms the portal vein system passing into the liver, the antigens and inflammatory factors from intestinal and splenic blood can enter the liver [24,25]. In addition, the gut and liver communicate via a tight bidirectional connection through the biliary tract [26]. As the second line of defense, the liver can eliminate invading bacteria and bacterial toxins, inhibiting the spread of hazardous substances into the systemic circulation [27]. Disruption of the gut–liver axis breaks the balance between immune activation and tolerance. The subsequent immune dysfunction exacerbates the pathogenesis and development of liver diseases.

Some animal studies have demonstrated that *P. gingivalis* is involved in the progression of non-alcoholic fatty liver disease (NAFLD) [28–30]. It has also been reported that *P. gingivalis*-derived virulence factors can cause excessive hepatic lipid accumulation and inflammatory reaction [31]. Besides, injection of *P. gingivalis* was found to induce immune response in the spleens of mice [32,33]. However, there has been inadequate research on the immune response to periodontal pathogens in spleen and liver via the oral–gut axis. The findings in the current study showed that repeated swallowing of *P. gingivalis* resulted in a change of intestinal microbiota and induction of the immunoinflammatory response in the gut, spleen, and liver. Our results provide a fresh perspective on the potential causal mechanisms by which periodontitis increases the risk of hepatic and systemic inflammation via the oral–gut axis.

## Materials and methods

### Bacterial cultures

The *P. gingivalis* W83 strain was provided by the Department of Oral Biology, China Medical University. The bacteria were inoculated on brain heart infusion agar with hemin (5 μg·mL^−1^), vitamin K (1 μg·mL^−1^), and 5% defibrinated sheep blood. This culture was then incubated at 37°C under anaerobic conditions (80% N_2_, 10% H_2_, 10% CO_2_) for approximately 6 days. Subsequently, colonies were streaked on a fresh medium plate for pure culture and then subjected to liquid enrichment for 16–18 h. Approximately 10^9^ colony-forming units (CFUs) is equivalent to an optical density of 1.0, and the optical density was read on an ultraviolet spectrophotometer (Spectrumlab 752Pro; Lengguang Technology Co., Ltd., Shanghai, China) at a wavelength of 600 nm.

### Mice

Six-week-old male C57BL/6 mice were provided by Liaoning Changsheng Biotechnology Co., Ltd. (Liaoning, China). The mice were raised under specific-pathogen-free conditions and fed regular chow and sterile water ad libitum during the experimental period. All animal procedures complied with the Guide for the Care and Use of Laboratory Animals of China Medical University (permit number 2,018,078).

### Oral administration

After an acclimation period of 1 week, the mice were randomized into two groups: a treatment group (SC-*P. g*) and a control group (SC-PBS). A total of 1 × 10^9^ CFUs of viable *P. gingivalis* was resuspended in 200 μL of phosphate-buffered solution (PBS). In the SC-*P. g* group, this suspension was given to each mouse every day for 9 weeks. The SC-PBS group underwent sham administration without the *P. gingivalis*. In the experiment, the daily performance and body weight of the mice were recorded every day.

### Illumina PE250 sequencing and bioinformatic analysis

Cecal contents were obtained and immediately frozen at −80°C for analysis of the microbial community after the 9 weeks of treatment. Total genome DNA from the samples was extracted using the CTAB/SDS method. DNA concentration and purity were monitored on 1% agarose gels. The DNA extracts of the samples were used to amplify the hypervariable regions V4-V5 of the *16S rRNA* genes, carried out in 30 µL reactions with 15 µL of Phusion®High-Fidelity PCR Master. Then, the PCR products was quantified, qualified (samples with bright main strip between 400–450 bp were chosen) and purified with the GeneJET Gel Extraction Kit (Thermo Scientific). Prior to high-throughput sequencing, a DNA library was prepared. Sequencing libraries were generated using the NEB Next®Ultra™DNA Library Prep Kit for Illumina (NEB, MA) following the manufacturer’s recommendations and index codes were added. At last, the library was sequenced on an Illumina MiSeq platform and 250 bp/300 bp paired-end reads were generated. Sequences analysis were performed by Usearch drive5. Sequences with ≥ 97% similarity were assigned to the same OTUs. The RDP classifier was used to annotate taxonomic information for each representative sequence. The alpha and beta diversities calculations were performed using Mothur software and R vegan package. A LEfSe analysis with non-parametric factorial Kruskal–Wallis sum-rank test was used to detect features with significantly different abundances between assigned taxa and to perform LDA to estimate the effect size of each feature. Sequencing and bioinformatics analyses were performed by Biozeron Technology Co. (Shanghai, China).

### Reverse-transcription quantitative polymerase chain reaction (RT-qPCR)

Total RNA extraction was performed using the RNAiso Plus Reagent Kit (9108Q; Takara Biotechnology Co., Ltd., Japan) and reverse-transcribed into complementary DNA (cDNA) using the PrimeScript^TM^ RT Reagent Kit with gDNA Eraser (RR047B; Takara Biotechnology Co., Ltd.). We used the SYBR1 Premix Ex Taq^TM^ II Reagent Kit (RR820B; Takara Biotechnology Co., Ltd.) to perform the real-time PCR reactions and used the QuantStudio™ Design & Analysis Software to conduct the real-time PCR analyses. The following PCR amplification conditions were used: initial denaturation at 95°C for 30s, followed by 40 cycles at 95°C for 5 s and 60°C for 34s. Specificity was confirmed by dissociation curve analysis. Amplification of each gene was repeated at least three times. Target RNA levels were normalized to the level of glyceraldehyde 3-phosphate dehydrogenase (GAPDH) mRNA. Table 1 shows the nucleotide sequences of the specific primer sequences.

**Table 1.** Primer sequences in RT-PCR

	Forward	Reverse
GAPDH	TGTGTCCGTCGTGGATCTGA	TTGCTGTTGAAGTCGCAGGAG
IFN-γ	CCAAGTTTGAGGTCAACAACCC	CGAATCAGCAGCGACTCCTT
TNF-α	TCCCAGGTTCTCTTCAAGG	CTGGTATGAGATAGCAAATCGG
IL-12a	TGCCTTGGTAGCATCTATGAGG	CGCAGAGTCTCGCCATTATGAT
IL-18	GACTCTTGCGTCAACTTCAAGG	CAGGCTGTCTTTTGTCAACGA
IL-21	CAGGCTAAGAGCTTGTATCGTTTGG	AGGACTGGCTGAGTCTTGAGCAC
IRF-1	GTTGTGCCATGAACTCCCTG	GTGTCCGGGCTAACATCTCC
CD3	CCTGAAAGCTCGAGTGTGTGAGTA	GATGGGCTCATAGTCTGGGTTG
B220	GTTATCCACGCTGCTGCCTCAC	TTGGCTGCTGAATGTCTGAGTGTC
F4/80	TGACTCACCTTGTGGTCCTAA	CTTCCCAGAATCCAGTCTTTCC
ROR-γt	GCCGCGGAGCAGACACACTT	GGAGGCCCCCTGGACCTCTG
Foxp3	CAGGAGAAAGCGGATACCAAATG	ATCTGTGAGGACTACCGAGCC
IL-17	TCAGCGTGTCCAAACACTGAG	CGCCAAGGGAGTTAAAGACTT
IL-6	ACAACCACGGCCTTCCCTACTT	CACGATTTCCCAGAGAACATGTG

### Western blotting

Total proteins of spleen and liver segments were isolated using radio immunoprecipitation assay (RIPA) lysis buffer (Beyotime Biotechnology, China) supplemented with phosphatase inhibitor buffer (1:100). The protein concentration was quantified with the BCA Protein Quantification Kit (Beyotime Biotechnology, China). Subsequently, equal amounts of protein (35–40 μg) were loaded on the gel together with 4 µL of protein ladder and moved onto polyvinylidene difluoride membranes electrophoretically. Then membranes were immersed in 5% skimmed milk powder in PBS at room temperature for 1 h. Subsequently, the membranes were incubated with primary antibodies at 4°C overnight. The dilutions of primary antibodies were as follows: β-actin at 1:1,000, IFN-γ at 1:1,000, STAT1 at 1:1,000, p-STAT1 at 1:1,000, STAT3 at 1:1,000, p-STAT3 at 1:1,500, IRF-1 at 1:1,000, ROR-γt at 1:2,000, Foxp3 at 1:1,000, F4/80 at 1:1,000, and CD3 at 1:1,000. After washing three times with PBST containing 0.05% Tween-20 the next day, the membranes were exposed to the matched secondary antibody (goat anti-rabbit IgG at 1:1,000) for 1 h. The Odyssey CLx Infrared Scanner (LI-COR, Lincoln, NE) was used to visualize the immunoreactive bands on membranes. For densitometry analysis, images were examined using ImageJ software.

### Organ indices and Lee’s index

Organ indices was used to represent the degree of damage and Lee’s index to evaluate the degree of obesity. Livers and spleens of the sacrificed mice from each group were obtained and impurities observed on the surface of the viscera were removed with filter paper. The liver and spleen were weighed with precision electronic weighing scales. Liver and spleen index was calculated as follows:

Organ indices = Organ weight(g)/Body weight(g) × 100

Lee’s index = [Weight (g) × 1000] ^(1/3)/^Length (cm) (Length refers to the distance from the tip of the nose to the anus.)

### Histological analysis

After the mice had been sacrificed, colon and liver tissues were separated and rapidly fixed in 4% neutral buffered formalin solution, embedded in paraffin, and then cut into 4-μm-thick paraffin sections. Oil Red O staining and hematoxylin and eosin (H&E) staining were carried out. The specimens were examined under a light microscope. The lipid area was calculated using the color-based thresholding plugin of the ImageJ software.

### Determination of aspartate aminotransferase (ALT), alanine aminotransferase (AST), and triglyceride (TG) in serum

In accordance with the manufacturer’s protocols, the activation levels of serum ALT, AST, and TG in mice were tested using commercial assay kits (IDEXX Laboratories, Inc., Westbrook, ME). The levels of ALT, AST, and TG are reported in U/L.

### Statistical analysis

Statistical analysis was carried out using the GraphPad Prism software (version: 8.0; GraphPad Software Inc., San Diego, CA). Values are presented as mean ± standard deviation (SD). According to the normal distribution, parametric or non-parametric tests were performed using unpaired *t*-test or Mann-Whitney test was used to determine the differences between the two independent groups. *P* values < 0.05 were considered to be statistically significant.

## Results

### Immune responses to P.gingivalis in the gut

#### Changes in gut microbiota after P.gingivalis administration

**Figure 1.**
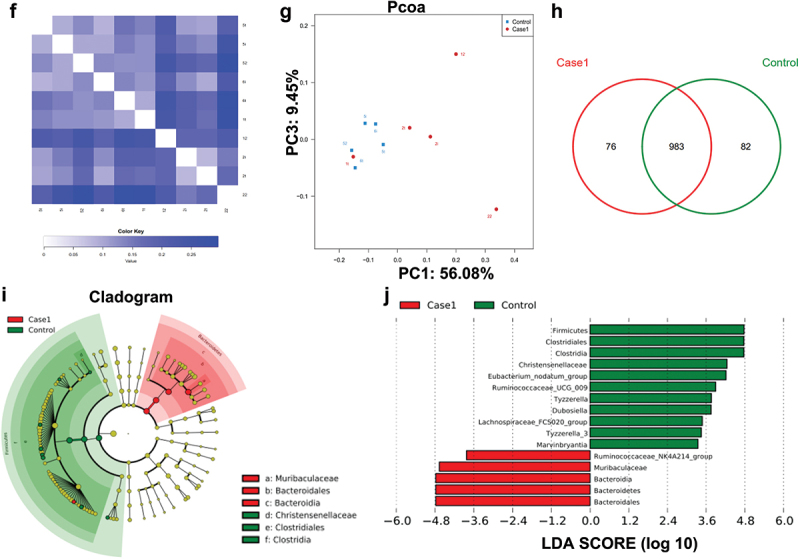
Analysis of the intestinal microbiota in the SC-PBS group and SC-*P. g* group. (n = 5). **(A–E)** Alpha diversity of the intestinal microbiota, including the diversity estimators (Shannon, Shannon-Wiener curve, and coverage) and richness estimators (Chao1 and rank-abundance distribution curve). **(F, G)** Beta diversity of the intestinal microbiota, including the distance heatmap and principal coordinates analysis. **(H–J)** Species composition and diversity, including Venn diagram of common and unique OTUs, cladogram, and linear discriminant analysis (LDA) score. * indicates a significant difference (*P* < 0.05). control: SC-PBS; case 1: SC-*P. g*. (two-tailed unpaired *t*-test).

The bacterial diversity was significantly decreased in SC-*P. g* mice (4.732 ± 0.176), compared with that in SC-PBS mice (5.017 ± 0.169, *P* < 0.05) (Figure 1 A and D). However, no difference in bacterial abundance was found between SC-*P. g* mice (897.358 ± 111.104) and SC-PBS mice (939.943 ± 45.941, *P* > 0.05) (Figure 1B). The species distribution in SC-PBS mice was more uniform than that in SC-*P. g* mice (Figure 1E).
**Figure 1.**
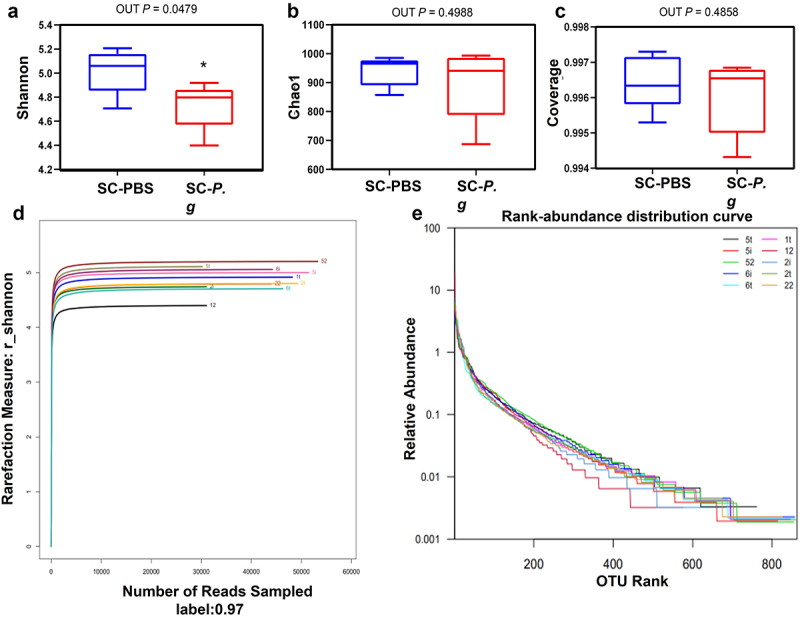
(continued).

The difference in the distribution of species abundance was greater in the SC-*P. g* mice than that in SC-PBS mice (Figure 1F). The microbiota structure of SC-*P. g* mice was different from that of SC-PBS mice, and the heterogeneity among samples of SC-*P. g* mice was greater (Figure 1G). From further analysis of the species distribution, 76 OTUs were found to be unique to SC-*P. g* mice and 82 were to SC-PBS mice (Figure 1H). At the phylum level, the proportion of *Bacteroidetes* in SC-*P. g* mice was significantly increased, while the proportion of *Firmicutes* was significantly decreased (*P* < 0.05). At the class level, the proportion of *Clostridia* was markedly decreased in SC-*P. g* mice (*P* < 0.05). At the order level, the proportion of *Clostridium* in SC-*P. g* mice was clearly decreased (*P* < 0.05). At the family level, the proportion of *Muribaculaceae* in SC-*P. g* mice was increased, while the proportion of *Christensenaceae* was decreased (*P* < 0.05). At the genus level, the proportion of *Ruminococcaceae*_NK4A214_group was increased in SC-*P. g* mice, whereas the proportions of *Eubacterium*_*nodatum_*group, *Ruminococcaceae*_U-CG_009, *Tyzzerella, Dubosiella, Lachnospiraceae*_FCS020_group, *Tyzzerella*_3, and *Marvinbryantia* were decreased (*P* < 0.05) (Figure 1I and J).

#### Intestinal inflammation after P.gingivalis administration

**Figure 2.**
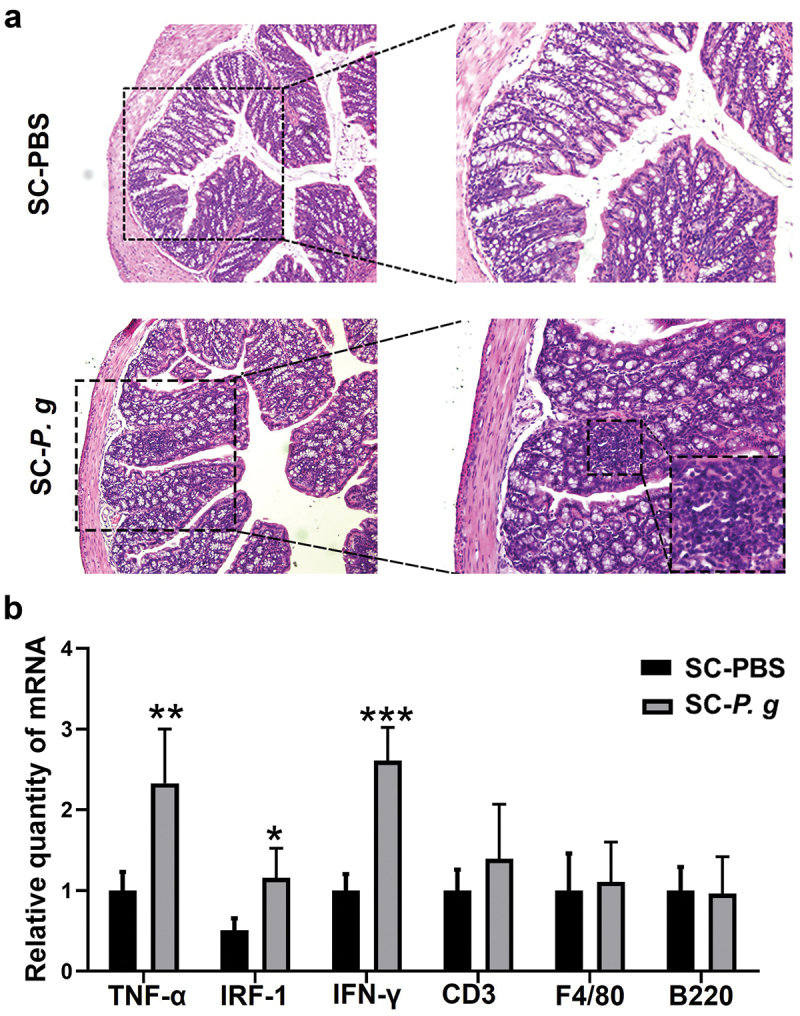
Analysis of intestinal inflammation in the SC-PBS group and SC-*P. g* group. (n = 5). **(A)** Representative images of colonic tissue stained by H&E. The images were taken at the magnification of 100× and 200 × . **(B)** mRNA expression of inflammatory factors in the colon. *, **, and *** indicate significant differences of *P* < 0.05, *P* < 0.01, and *P* < 0.001 compared with the SC-PBS group, respectively. (two-tailed unpaired *t*-test).

H&E staining of colonic tissue showed no significant differences in goblet cell morphology and intestinal mucosal structure between the SC-PBS group and SC-*P. g* group. The infiltrated inflammatory cells in lamina propria were significantly more increased in the SC-*P. g* mice than in SC-PBS mice (Figure 2A).

The RT-qPCR data indicated that the mRNA levels of the tumor necrosis factor (TNF)-α, interferon regulatory factor (IRF)-1, and interferon (IFN)-γ in colonic tissue of SC-*P. g* mice were significantly increased (TNF-α: *P* < 0.01; IRF-1: *P* < 0.05; IFN-γ: *P* < 0.001) (Figure 2B), while the expression of CD3, F4/80, and B220 genes showed no marked change between the two groups (Figure 2B).

### Immune responses to P.gingivalis in the spleen

**Figure 3.**
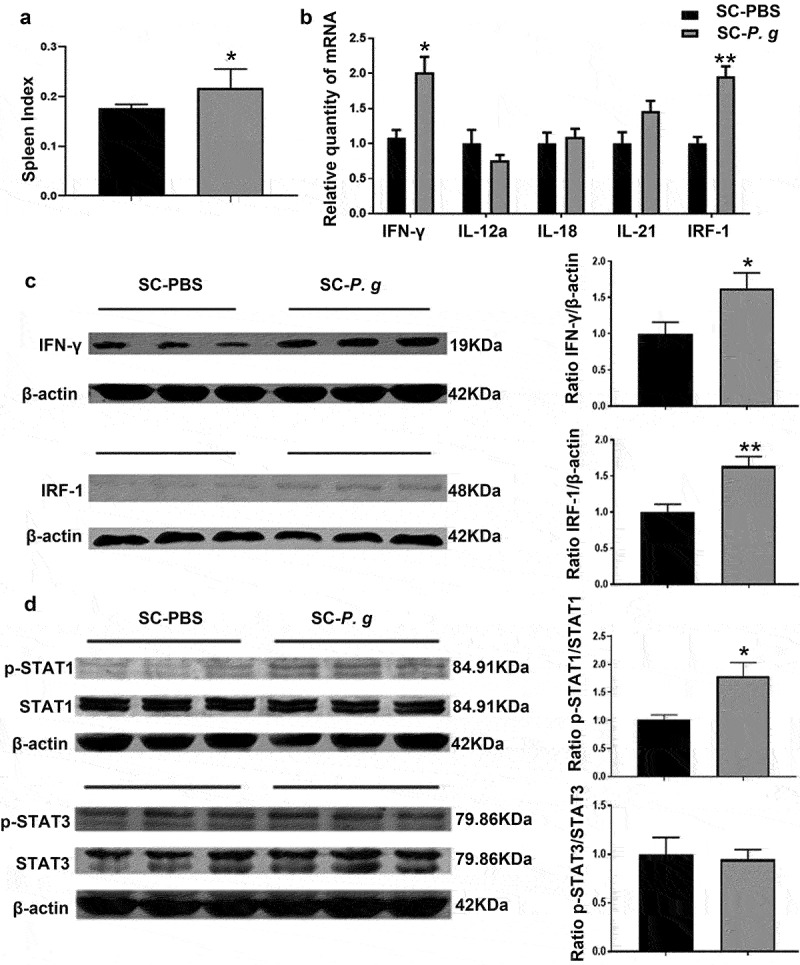
Analysis of splenic inflammation in the SC-PBS group and SC-*P. g* group. (n = 5). **(A)** the spleen index. **(B)** mRNA expression of inflammatory factors in the spleen. **(C)** protein levels of IFN-γ and IRF-1 in the spleen. **(D)** phosphorylation levels of STAT1 and STAT3 in the spleen. * and ** indicate significant differences at *P* < 0.05 and *P* < 0.01 compared with SC-PBS, respectively. (two-tailed unpaired *t*-test).

Compared with that in the SC-PBS group, the spleen index was markedly higher in the SC-*P. g* group (*P* < 0.05) (Figure 3A). mRNA and protein levels of IFN-γ and IRF-1 were upregulated in the spleen samples of the SC-*P. g* mice (IFN-γ: *P* < 0.05; IRF-1: *P* < 0.01) (Figure 3 B and C). The levels of activation (reflected by phosphorylation) of signal transducer and activator of transcription (STAT)1 and STAT3, downstream signaling molecules of the IFN-γ pathway, were also examined. The results proved that the phosphorylation level of STAT1 in the spleen of SC-*P. g* mice was increased (*P* < 0.05), but no significant difference in the phosphorylation level of STAT3 was found (Figure 3D). According to the complete blood cell count analysis, the counts of white blood cells, lymphocytes and neutrophils were significantly increased in the blood of the SC-*P. g* mice (Figure S1).

### *Immune responses to P.gingivalis* in the liver

#### Inflammatory changes in the liver after P.gingivalis administration

**Figure 4.**
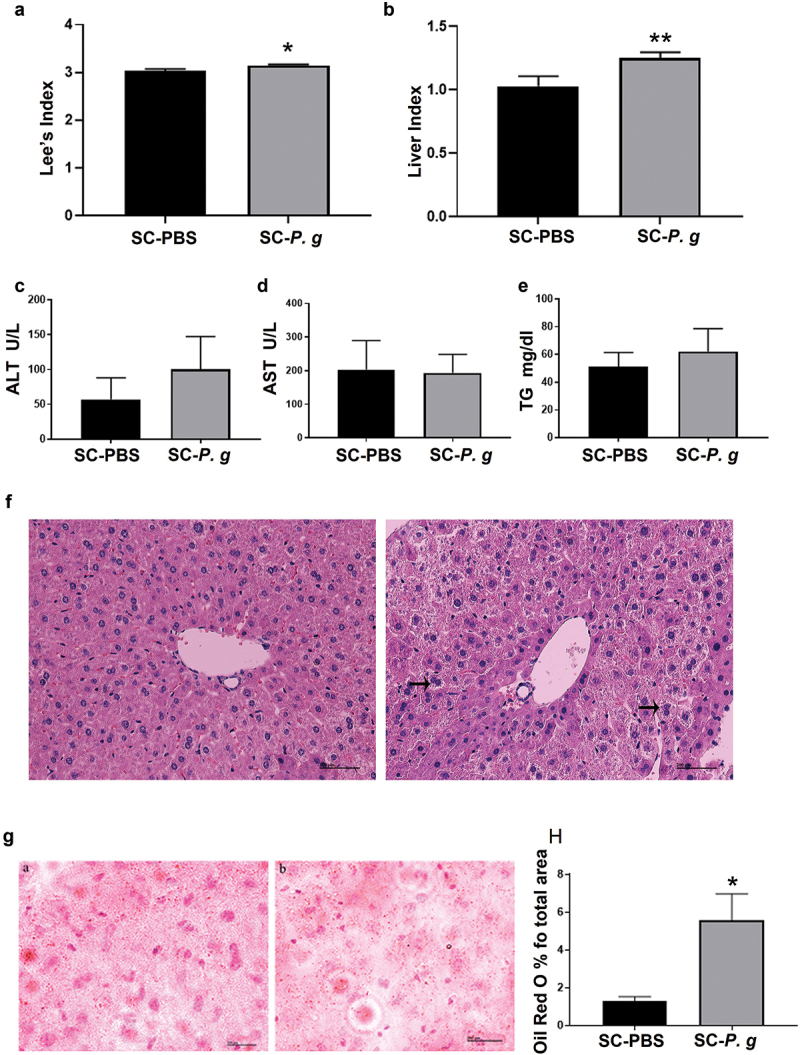
Analysis of hepatic inflammation in the SC-PBS group and SC-*P. g* group. (n = 5). **(A, B)** Lee’s index and liver index. **(C-E)** The levels of ALT, AST, and TG in serum. **(F)** Representative images of liver tissue stained by H&E. The images were taken at the magnification of 200 × (scale bar, 200 µm). Black arrows indicate ballooning degeneration. **(G)** Representative images of liver tissue with Oil Red O staining. The images were taken at the magnification of 200 × (scale bar, 200 µm). **(H)** lipid (%) were quantified by the percent of lipid area to total area and performed by ImageJ software. Statistical analyses were performed using the Mann-Whitney *U* test (A, E, and H) and two-tailed unpaired *t*-test (B, C, and D). * and ** indicate significant differences at *P* < 0.05 and *P* < a 0.01 compared with SC-PBS, respectively.

Compared with those in the SC-PBS group, Lee’s index (*P* < 0.05) and liver index (*P* < 0.01) were significantly higher in the SC-*P. g* group (Figure 4 A and B). AST, ALT, and TG were detected, and the levels of AST and TG showed increasing trends in serum of SC-*P. g* mice, but without statistical significance (Figure 4 C-E). Upon analyzing the H&E staining of liver tissue, there was no steatosis in the liver tissue of SC-PBS mice, with no tissue damage. The liver lobules were intact and cells were distributed radially around the central vein. By contrast, some of the hepatocytes were structurally disordered in the liver tissue of SC-*P. g* mice (Figure 4F). In addition, Oil Red O staining showed that steatosis was more extensive in the liver tissue of SC-*P. g* mice than in that of SC-PBS mice (*P* < 0.05) (Figure 4 G and H).

#### Changes of hepatic immune cells after P.gingivalis administration

**Figure 5.**
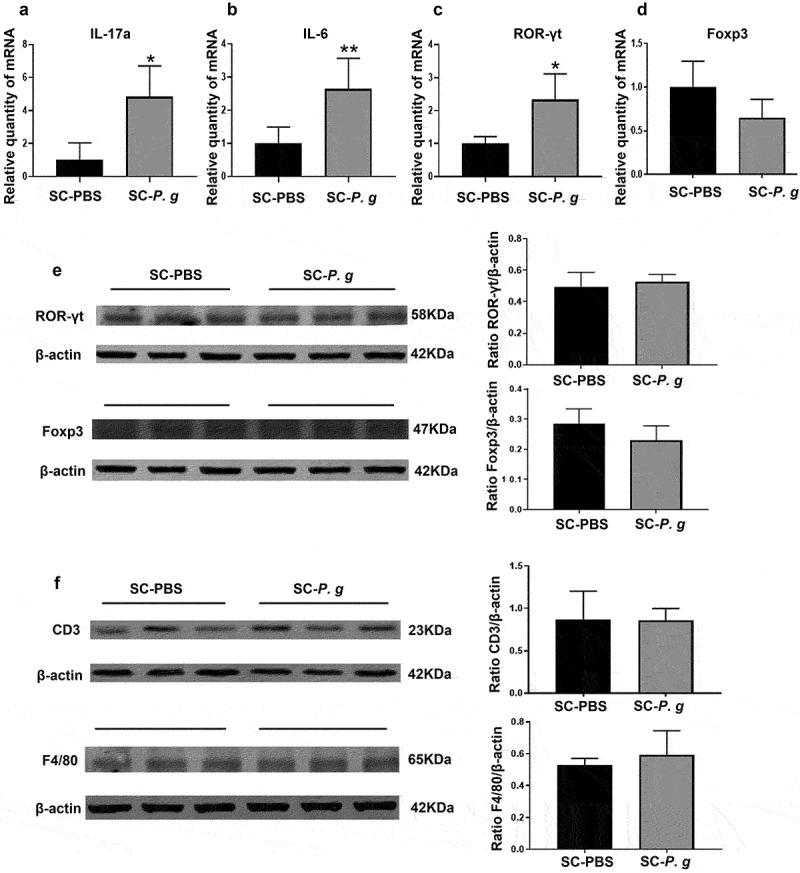
Analysis of hepatic inflammatory cell markers in the SC-PBS group and SC-*P. g* group. (n = 5). **(A-D)** mRNA expression of inflammatory markers in liver tissue in mice. **(E, F)** Protein levels of inflammatory cell markers in liver. Statistical analyses were performed using the Welch’s *t*-test (A) and unpaired *t*-test (B-F). * and ** indicate significant differences at *P* < 0.05 and *P* < 0.01 compared with SC-PBS, respectively.

The RT-qPCR results revealed that interleukin (IL)-17a, IL-6 and retinoic acid receptor-related orphan nuclear receptor gamma t (ROR-γt) were upregulated and the forkhead/winged-helix family transcriptional repressor p3 (Foxp3) was downregulated in the liver tissues of SC-*P. g* mice (IL-17a and ROR-γt: *P* < 0.05; IL-6: *P* < 0.01) (Figure 5 A-D). However, the protein expression levels of ROR-γt, Foxp3, F4/80, and CD3 showed no changes between the SC-*P. g* group and SC-PBS group (Figure 5 E and F).

## Discussion

The results of the current study demonstrated the observable alteration of the gut microbiota and inflammation in gut, spleen, and liver following the oral administration of *P. gingivalis*. In the gut and spleen, a predominant Th1 response was evident and inflammatory responses were present in the liver.

The intestinal microbiota, as the biological barrier of the gut, performs certain basic functions [20]. Accumulating evidence has shown that disturbance of the intestinal microbiota is closely related to various diseases, such as IBD and NAFLD [34]. Invasion of foreign pathogenic bacteria is one of the most important factors disrupting the intestinal microbiota. In this study, the intestinal microbiota was found to be significantly changed in SC-*P. g* mice, with a decrease in bacterial diversity, which is known to be associated with intestinal inflammation and hepatic diseases [35]. *Bacteroidetes* and *Firmicutes* are the dominant microbiota in the gut [36]. Here, the ratio of *Bacteroidetes* to *Firmicutes* was found to be increased in SC-*P. g* mice, which is consistent with the literature [17,37]. In hepatic diseases, the proportion of *Firmicutes* was reported to be decreased and that of *Bacteroidetes* was increased [38,39]. The gut microbes, their components or their metabolites, e.g. short-chain fatty acids (SCFAs), trimethylamine and bile acids, generated in the gut can enter the circulation and effectively launch an inflammatory response on both a local and systemic scale [40]. *Ruminococcaceae, Lachnospiraceae*, and *Marvinbryantia* boost the production of SCFAs [41–43]. *Clostridium* and *Eubacterium* participate in the conversion of primary bile acids into secondary bile acids. The family *Christensenellaceae* is significantly related to individual variation in the body mass index and to blood levels of TG and high-density lipoprotein [44]. Our findings demonstrated that the relative abundances of the above bacterial species were decreased in SC-*P. g* mice, which might have caused the intestinal and systemic inflammation.

Inflammatory infiltration in the gut is also an important pathologic change induced by oral administration of *P. gingivalis*. The mRNA levels of TNF-α, IFN-γ, and IRF-1 were elevated in the gut of SC-*P. g* mice. TNF-α and IFN-γ are canonical Th1 cytokines. TNF-α, as a pleiotropic cytokine, has been shown to correlate with numerous intestinal diseases such as active celiac disease and IBD [45,46]. Some studies have also demonstrated the overexpression of intestinal TNF-α in patients with Crohn’s disease [47–49], and anti-TNF-α antibody treatment has been used to treat patients with IBD [50,51]. In mucosal tissues of murine colitis models and IBD patients, the level of IFN-γ is highly upregulated. The pathophysiological role of IFN-γ in mucosal tissues of IBD has been ascribed to its immunomodulatory or epithelial effects [52,53]. IRF-1, the first member of the IFN regulatory factor family, exerts effects in various physiological and pathological contexts including inflammatory injury, viral infection, development of the immune system, and autoimmunity [54]. We demonstrated the induction of IRF-1 mRNA expression in the gut of SC-*P. g* mice, consistent with IFN-γ-initiated signaling events. Thus, orally administered *P. gingivalis* markedly affected the intestinal physiological environment of mice in the current study.

Disorders of the intestinal microbiota and immune response are often accompanied by impaired intestinal barrier function. We examined the expression levels of tight junction proteins in the colon but found no changes (data unpublished). We speculated that *P. gingivalis* might induce abnormal distribution of tight junction proteins rather than their content change. Further studies are needed to clarify the effect of *P. gingivalis* on the colonic barrier. In addition to the alteration of the gut microbial composition by swallowed *P. gingivalis*, proposed mechanisms for swallowed *P. gingivalis* inducing intestinal inflammation have also included the direct effect of *P. gingivalis* on the gut. *P. gingivalis* can disrupt the innate immune and inflammatory responses using a range of virulence factors [55]. However, relevant studies are still few and the mechanism is inconclusive in the gut. It remains to be elucidated whether a direct cause-and-effect relationship exists between *P. gingivalis* and immune response in the gut.

The spleen maintains peripheral tolerance and modulates the immune system by clearing circulating apoptotic cells and promoting the differentiation and activation of T and B cells [56]. Its pathological changes mostly reflect the activation of systemic inflammation [57,58]. Evidence has shown that hepatic and splenic activities are linked to immunity, infections, and metabolism [59]. In our study, the expression levels of inflammatory cytokines were markedly upregulated in the spleen, and the IFN-γ/STAT1 pathway was abnormally activated. STAT1, a signal transduction protein, modulates the biological function of IFN-γ. Cytokines such as IFN-γ activate STAT1 by inducing the phosphorylation of one of its tyrosine residues (Tyr701) [60]. After forming a homodimer or heterodimer, p-STAT1 translocates from the cytoplasm to the nucleus and binds to gene promoters containing specific binding elements to regulate the expression of interferon-stimulated genes (ISGs) [61,62]. Abnormal activation of the IFN-γ/STAT1 pathway has made it a focus of research on inflammatory diseases [63]. As a member of the ISGs, IRF-1 is a downstream transcription factor in the IFN-γ/STAT1 pathway. Researchers have found that IFN-γ/STAT1 signaling can inhibit the differentiation and function of Treg cells, in which IRF-1 may exert important effects [64]. Because the spleen is considered to be a peripheral immune organ, splenic inflammation means that the systemic immune response is activated. The conclusion was further supported by the increased blood cell count of lymphocytes and neutrophils (Figure S1), higher organ indices (including those of liver and spleen) and higher Lee’s index in *P. gingivalis*-administered mice.

As the central metabolic hub, the liver regulates the balance between nutritional inputs and metabolic outputs. AST and ALT are important amino acid transaminases in the body, the plasma levels of both of which are sensitive indicators that can reflect damage to liver cells [65]. Besides, the accumulation of TG has been implicated in the pathogenesis of insulin resistance and could act as a metabolic marker in the liver [66]. In our study, the AST and TG plasma levels were slightly increased in SC-*P. g* mice, albeit not significantly. A previous study using multivariable linear regression analyses found that periodontitis is significantly associated with serum AST and ALT levels in healthy Japanese women (20–59 years old) [67]. In another previous study, a significant link between ALT level (68.5 ± 9.4 years old) and serum IgG antibody titer against *P. gingivalis* was identified in women, but not in men [68]. Moreover, in patients with NAFLD, no significant differences in serum ALT and AST levels were observed between patients with and without *P. gingivalis* detected in saliva [69]. There are inconsistent findings regarding the changes of AST, ALT, and TG in the published literature, possibly due to multiple factors such as sex hormones and age.

In the present study, the results of H&E staining and Oil Red O staining showed hepatocyte ballooning and lipid accumulation in SC-*P. g* mice. Abnormal lipid accumulation and hepatocyte ballooning are pathological characteristics of hepatic steatosis, which can develop into NAFLD and nonalcoholic steatohepatitis in severe cases. Multiple factors such as alterations to the intestinal microbiota, gut barrier dysfunction, and obesity contribute to hepatic steatosis and inflammation [70]. We proved that *P. gingivalis* induced the disruption of intestinal homeostasis and led to weight gain (data not shown), which could trigger hepatic steatosis. Treg cells and Th17 cells are two distinct subpopulations of Th1 and Th2 cells, which exert opposing effects on immune reactions [71]. The maintenance of an appropriate balance between anti-inflammatory Tregs and pro-inflammatory Th17 cells may be important for immune homeostasis. Destabilization of this equilibrium may give rise to autoimmunity and chronic inflammation [72]. Th17 cells are commonly reported as the major IL-17a-secreting cells [73]. IL-17a is a critical proinflammatory and tumor promoting cytokine that regulates de novo lipogenesis and chemokine production in metabolically injured hepatocytes. The overactivation of the IL-17 axis could facilitate the liver damage in diverse contexts [74,75]. IL-6, a key immunomodulator, participates in the differentiation of naïve or Treg cells into Th17 cells. IL-6 acts synergistically with TGF-β to induce the expression of the transcription factor ROR-γt, promoting Th17 differentiation [76,77]. In our study, the gene expression levels of IL-17a, IL-6, and ROR-γt in the liver were significantly increased in SC-*P. g* mice. The elevation of IL-17a and IL-6 induced by orally administrated *P. gingivalis* might activate the inflammatory response in liver. However, we did not observe changes in the levels of ROR-γt and Foxp3 proteins, probably because the young mice used in this study had strong immunity against bacterial infection. Clinically, patients with periodontal disease are typically middle-aged or elderly, with compromised immunity. In the current study, the subtle equilibrium of Th17/Treg cells was skewed toward Th17 cells. *P. gingivalis* might thus disrupt the Th17/Treg balance, making the liver susceptible to inflammation. Our data confirmed that orally administered *P. gingivalis* could induce hepatic inflammatory injury, which drives the progression of hepatic diseases.

Although animal models used so far do not perfectly recapitulate patients with periodontitis, they have led to the discovery of important concepts in its pathogenesis. At present, experimental animal models can be made by oral inoculation, oral gavage or intraperitoneal injection of *P. gingivalis* or its virulence factors to study the pathogenic effects of periodontal pathogens on digestive system diseases. In view of the close relationship between the oral cavity and gut anatomically and physiologically, we believe that it is a good choice to model the intestinal environment of patients with periodontitis by using oral gavage in mice.

Of course, the present study has some limitations that could be addressed in future research. In this trial, we focused mainly on the immune response induced by *P. gingivalis* in the colon, but not the small intestine, aiming to explore the potential link between periodontitis and IBD. Because the small intestine plays a major role in digestion and has a close connection with the liver anatomically and physiologically [78,79], it is most likely to be involved in the extra-intestinal and systemic immunoinflammatory responses induced by periodontal pathogens. Therefore, further investigation is needed to verify this conjecture. In addition, enlarging the sample size should also be an important consideration in future research.

In summary, the current study demonstrated disordered gut microbiota and disequilibrium of immune responses in gut, spleen, and liver caused by orally administered *P. gingivalis* in mice. The results also showed that Th1 cells and Th1-related molecules (IFN-γ and TNF-α) in gut and spleen were increased upon *P. gingivalis* administration. However, the exact mechanisms behind these findings have yet to be clarified. Our results may provide a fresh perspective on the effects of oral pathogens on systemic inflammation. The findings might also lead to therapeutic and preventive targets in the treatment of systemic inflammation.

## Supplementary Material

Supplemental Material
