# Supplementary material for: Inflammatory response of gut, spleen, and liver in mice induced by orally administered Porphyromonas gingivalis
Source: J Oral Microbiol. 2022 Jun 16;14(1):2088936. doi: 10.1080/20002297.2022.2088936 (PMC9225697; doi:10.1080/20002297.2022.2088936)
Supplement: Supplemental Material [file ZJOM_A_2088936_SM0675.docx]

| Parameter/Group | SC±PBS | SC±*P. g* |
| --- | --- | --- |
| WBC (K/μL) | 1.220±0.257 | 2.956±0.619^***^ |
| NEUT (K/μL) | 0.150±0.067 | 0.268±0.069^*^ |
| LYMPH (K/μL) | 1.060±0.199 | 2.676±0.553^***^ |
| MONO (K/μL) | 0.008±0.004 | 0.012±0.004 |
| EO (K/μL) | 0.002±0.004 | 0.004±0.005 |
| BASO (K/μL) | 0 | 0.004±0.005 |
| NEUT (%) | 11.560±4.250 | 9.960±1.378^*^ |
| LYMPH (%) | 87.560±4.083 | 90.540±0.939 |
| MONO (%) | 0.700±0.415 | 0.500±0.141 |
| EO (%) | 0.180±0.360 | 0 |
| BASO (%) | 0 | 0.200±0.276 |
| RBC (M/μL) | 10.944±0.457 | 11.256±0.225 |
| HGB (g/dL) | 15.580±0.445 | 15.800±0.316 |
| HCT (%) | 53.780±2.516 | 54.880±1.155 |
| MCV (fL) | 49.140±0.372 | 49.080±0.402 |
| MCH (pg) | 14.160±0.120 | 14.080±0.040 |
| MCHC (g/dL) | 28.700±0.525 | 28.880±0.426 |
| RDW±CV (%) | 24.060±0.703 | 24.240±0.224 |
| PLT (K/μL) | 611.600±172.039 | 660.400±195.731 |
| MPV (fL) | 7.160±0.480 | 7.260±0.314 |
| PDW (fL) | 8.580±0.496 | 9.040±0.589 |
| PCT (%) | 0.452±0.117 | 0.476±0.127 |

**FigureS1**. The results of complete blood count. WBC , white blood cells; NEUT, neutrophils; LYMPH, lymphocytes; MONO, monocytes; EO, eosinophils; BASO, basophils; RBC, red blood cells; HGB, hemoglobin; HCT, hematocrit; MCV, mean corpuscular volume; MCH, mean corpuscular hemoglobin; MCHC, mean corpuscular hemoglobin concentration; RDW±CV, red blood cell distribution width by coefficient of variation; PLT, platelets; MPV, mean platelet volume; PDW, platelet distribution width; PCT, plateletcrit. Values are presented as the mean ± standard deviation (n=5 mice/group). Statistical analysis was used by unpaired *t*±test. *, **, and *** indicate significant differences of *P* < 0.05, *P* < 0.01, and *P* < 0.001 compared with the SC±PBS group, respectively.
